# Photosensitizing Medications and Skin Cancer: A Comprehensive Review

**DOI:** 10.3390/cancers13102344

**Published:** 2021-05-12

**Authors:** Elisabeth A. George, Navya Baranwal, Jae H. Kang, Abrar A. Qureshi, Aaron M. Drucker, Eunyoung Cho

**Affiliations:** 1 Warren Alpert Medical School of Brown University, Providence, RI 02903, USA; elisabeth_george@brown.edu (E.A.G.); navya_baranwal@brown.edu (N.B.); 2 Channing Division of Network Medicine, Brigham and Women’s Hospital and Harvard Medical School, Boston, MA 02115, USA; nhjhk@channing.harvard.edu; 3 Department of Dermatology, Rhode Island Hospital and Warren Alpert Medical School of Brown University, Providence, RI 02903, USA; abrar_qureshi@brown.edu; 4 Department of Epidemiology, School of Public Health, Brown University, Providence, RI 02903, USA; 5 Division of Dermatology, Department of Medicine, University of Toronto, Toronto, ON M5S 3H2, Canada; aaron_drucker@brown.edu; 6 Women’s College Research Institute and Division of Dermatology, Department of Medicine, Women’s College Hospital, Toronto, ON M5S 1B2, Canada; 7 Department of Dermatology, Warren Alpert Medical School of Brown University, Providence, RI 02903, USA

**Keywords:** photosensitizing medication, melanoma, basal cell carcinoma, squamous cell carcinoma

## Abstract

**Simple Summary:**

Photosensitizing medications are commonly used in the United States, and studies have begun exploring whether exposure to these medications may be a risk factor for skin cancer. As the population in the United States ages, there are more elderly patients being prescribed photosensitizing medications to treat chronic conditions. At the same time, the incidence of skin cancer is increasing. We summarize current evidence regarding the risk of skin cancer associated with use of each type of photosensitizing medication and highlight gaps in the literature to guide further study.

**Abstract:**

(1) The incidence of skin cancer is increasing in the United States (US) despite scientific advances in our understanding of skin cancer risk factors and treatments. In vitro and in vivo studies have provided evidence that suggests that certain photosensitizing medications (PSMs) increase skin cancer risk. This review summarizes current epidemiological evidence on the association between common PSMs and skin cancer. (2) A comprehensive literature search was conducted to identify meta-analyses, observational studies and clinical trials that report on skin cancer events in PSM users. The associated risks of keratinocyte carcinoma (squamous cell carcinoma and basal cell carcinoma) and melanoma are summarized, for each PSM. (3) There are extensive reports on antihypertensives and statins relative to other PSMs, with positive and null findings, respectively. Fewer studies have explored amiodarone, metformin, antimicrobials and vemurafenib. No studies report on the individual skin cancer risks in glyburide, naproxen, piroxicam, chlorpromazine, thioridazine and nalidixic acid users. (4) The research gaps in understanding the relationship between PSMs and skin cancer outlined in this review should be prioritized because the US population is aging. Thus the number of patients prescribed PSMs is likely to continue to rise.

## 1. Introduction

Skin cancers, including basal cell carcinoma (BCC), squamous cell carcinoma (SCC) and melanoma, are the most common cancers in the United States (US), and incidence rates are increasing [1]. Invasive melanoma is the deadliest of these, due to its high metastatic potential. From 2011 to 2015, the incidence of cutaneous melanoma, which we will hereon refer to as melanoma, increased substantially with an average annual percent change of 2.3% in men and 1.7% in women [2]. The age-adjusted rates of new melanoma cases increased from 27.9 per 100,000 in 2011 to 29.9 per 100,000 in 2015 in men and from 16.5 per 100,000 in 2011 to 18.5 per 100,000 in 2015 in women [3]. US projections for 2020 estimate 100,350 new cases and 6850 new deaths due to melanoma [4]. Even though mortality rates of melanoma have been declining, mortality rates of keratinocyte carcinoma (KC), defined as BCC and SCC, have increased by 44% from 2011 to 2017 [5]. The decreasing mortality rates of melanoma may be attributed to novel therapeutics such as targeted agents and immune checkpoint inhibitors that have been implemented into standard treatment guidelines [6].

Prolonged and excessive exposure to ultraviolet (UV) radiation from natural and artificial sources are major risk factors for skin cancer [7,8]. Other prominent risk factors include constitutional factors such as light hair color, immunosuppression, skin that burns easily, exposure to heavy metals and a personal or family history of skin cancer [7,9,10]. Photosensitizing medications (PSMs) have been suggested as another potential risk factor for skin cancer [11,12]. Drug metabolites of phototoxic medications may interact with UV radiation resulting in the production of reactive oxygen species (ROS). These ROS directly damage cell membrane lipids, proteins and DNA, leading to cellular damage that may result in skin carcinogenesis [13]. Over 300 medications have been classified as phototoxic [14]. Use of PSMs is extremely common. For example, in Austria and Germany, it was estimated that about half of prescription medications were PSMs [15]. A case-control study in the Netherlands found that over 40% of the population used PSMs [16].

Herein, we provide a comprehensive review of the current epidemiological evidence on the association between commonly used PSMs and skin cancer. When appropriate, in vitro and animal studies are discussed to highlight biological mechanisms that may explain the epidemiological findings. Gaps in the literature are highlighted to guide future research directions.

## 2. Materials and Methods

We conducted a narrative review of the association between various phototoxic medications and skin cancer. Medications were selected based on a review article that highlights common PSMs, according to literature review and the authors’ experience [17]. We searched PubMed from inception to 25 October 2019 using the keywords “skin cancer”, “phototoxic” and the individual names of the PSMs. The search was not limited by language.

This review explored antihypertensives, such as diuretics, calcium-channel blockers (CCBs), beta-blockers, angiotensin-converting enzyme inhibitors (ACEi) and angiotensin receptor blockers (ARB), antidiabetic drugs, amiodarone, nonsteroidal anti-inflammatory drugs (NSAIDs), statins, voriconazole, antibacterial agents and vemurafenib (Table 1). These PSMs were investigated because there are epidemiological studies on their relation to skin cancer. We did not find any epidemiological studies on other common PSMs including glyburide, chlorpromazine, nalidixic acid and thioridazine and the risk of skin cancer.

We included case-control studies, cohort studies and randomized-controlled trials (RCT). If multiple meta-analyses on an individual medication and skin cancer were available, we chose the most recent or most comprehensive analysis and described these findings. We also described individual studies, which were not included in the meta-analyses. If a meta-analysis of the medication was not available, we summarized individual studies of the medication and skin cancer. For most medications, we describe the findings of melanoma and KC separately.

## 3. Results

### 3.1. Antihypertensives

#### 3.1.1. Diuretics

Since their introduction more than 60 years ago, diuretics have become a backbone for antihypertensive treatment regimens. Certain subtypes of diuretics have been linked to phototoxic reactions, including thiazides (i.e., hydrochlorothiazide and bendroflumethiazide), thiazide-like diuretics (i.e., chlorthalidone and indapamide), loop diuretics (i.e., furosemide and bumetanide) and potassium-sparing diuretics (i.e., amiloride, spironolactone and triamterene) [18]. The sulfonamide ring is the common structure that appears in thiazide diuretics, thiazide-like diuretics and loop diuretics, contributing to the photosensitizing properties of these drugs. UV radiation may induce dissociation of the chlorine substituent in the structure resulting in free radical reactions [19]. When activated by UV radiation, thiazide diuretics act as a chromophore and cause dimerization of pyrimidines within DNA [20,21].

##### Keratinocyte Carcinoma

Multiple meta-analyses [13,22,23,24,25] have investigated the relationship between use of diuretics, especially thiazide diuretics and skin cancer. The most recent meta-analysis found a small increased risk of BCC associated with thiazide use with summary relative risk (RR) of 1.17 (95% confidence interval [CI] 1.03–1.33), including two case-control studies, one matched cohort study and one cohort study [26]. The analysis for SCC (including five case-control studies, one matched cohort study and one RCT) found a stronger association (RR 1.93, 95% CI 1.59–2.35) with use of thiazide diuretics. Some of the included studies in the meta-analyses reported dose-response associations based on either dose or duration of thiazide use. Thiazide diuretics were evaluated as a drug class in some individual studies, while other studies assessed hydrochlorothiazide and bendroflumethiazide separately. Hydrochlorothiazide use was more consistently associated with KC compared to bendroflumethiazide use.

##### Melanoma

The meta-analysis also found that the RR of melanoma associated with thiazide diuretics use was 1.17 (95% CI 1.12–1.24), with a dose-response relationship based on three case-control studies and one matched cohort study [26]. The majority of included studies were based on Caucasian populations. A recent case-control study in Taiwan found no association between hydrochlorothiazide use and the risks of melanoma, lip KC and non-lip KC [27].

#### 3.1.2. CCBs

Certain CCBs may have photosensitizing properties similar to that of thiazide diuretics [15]. However, some T-type and L-type CCBs (e.g., verapamil and dihydropyridine-CCB) may reduce melanoma metastasis by inhibiting autophagy and promoting apoptosis. These types of CCB have been investigated in the treatment of advanced melanoma [28,29,30]. In a study of metastatic melanoma, a combined therapeutic regimen of verapamil, dacarbazine and interferon-alpha was better tolerated and more effective than dacarbazine monotherapy [30].

##### Keratinocyte Carcinoma

Literature on the risk of skin cancer among CCB users includes two meta-analyses [23,25]. Gandini et al. computed the risk of skin cancer overall (KC and melanoma) among CCB users based on nine studies, including a total of 50,655 skin cancer cases (six cohort studies, two RCTs and one case-control study) [23]. The risks of skin cancer and KC were moderately increased among CCB users (all skin cancers: RR 1.14, 95% CI 1.07–1.21; *KC*: RR 1.16, 95% CI 1.06–1.27). The study did not report separately on the risks of BCC versus SCC. Tang et al. included three studies (one case-control, two cohort) and found that CCB use was associated with small increased risk of BCC (odds ratio [OR] 1.15, 95% CI 1.09–1.21), but not with SCC (OR 1.03; 95% CI, 0.88–1.21) [25].

##### Melanoma

A meta-analysis including the results from five studies (three cohort, one RCT, one case-control) found a modestly increased risk of melanoma (RR 1.11, 95% CI 1.06–1.27) among CCB users [24].

#### 3.1.3. Beta-Blockers 

Tilisolol and nebivolol are beta-blockers that have been reported to cause photosensitive reactions [31,32]. On the other hand, certain beta-blockers such as carvedilol and propranolol may mediate cancer prevention and inhibit melanoma development, as demonstrated in animal, in vitro and in vivo studies [33,34,35,36,37,38].

Unfortunately, epidemiological studies have not evaluated the association between individual beta-blockers and skin cancer. Rather, the evidence has been presented with all beta-blockers grouped into a single category of exposure.

##### Keratinocyte Carcinoma

A meta-analysis based on one case-control and two cohort studies found that beta-blocker use was associated with modestly increased risk of BCC (OR 1.09, 95% CI 1.04–1.15) compared to non-use, but was not associated with the incidence of SCC (OR 0.89, 95% CI 0.69–1.16) [25]. The case-control study explored patients with first-time diagnosis of skin cancer, whereas the two cohort studies were focused on populations at high-risk for skin cancer including veterans and kidney transplant recipients. Another meta-analysis, which included two cohort studies, reported no association with risk of KC [23].

##### Melanoma

In a meta-analysis based on two case-control and two cohort studies, beta-blocker use was significantly associated with an increased risk of melanoma with RR of 1.21 (95% CI 1.05–1.40) [23]. Other epidemiological studies have evaluated beta-blockers as treatment for melanoma. A few of them did not find any beneficial role of post-diagnosis beta-blocker use and melanoma-specific mortality or all-cause mortality [39,40]. However, other studies found that beta-blocker users had a reduced recurrence of melanoma and better overall survival [41,42,43].

#### 3.1.4. Angiotensin-Converting Enzyme inhibitors (ACEi) and Angiotensin Receptor Blockers (ARB)

ACEi prevent angiotensin-converting enzyme (ACE) from converting angiotensin I to angiotensin II, whereas ARB prevent binding of angiotensin II to its receptor, angiotensin II type 1 receptor (AT1R) [44]. Angiotensin II signaling promotes VEGF-mediated angiogenesis, leading to increased proliferation of tumor cells [45,46,47]. In melanoma, the renin-angiotensin system has both tumor suppressor and oncogenic effects [48]. ACEi have been documented to cause phototoxicity by positive photopatch testing for ramipril and rechallenge evidence for quinapril [45]. A recent review of the WHO database demonstrated numerous phototoxic reactions to ARB, specifically losartan, irbesartan and valsartan [49]. Taken together, ACEi and ARB may exhibit both chemopreventive and oncogenic properties in skin carcinogenesis.

##### Keratinocyte Carcinoma

A meta-analysis on ACEi use and the risk of KC included eight studies (three cohort, one matched cohort, three case-control and one RCT) with 37,618 cases of skin cancer [23]. The RR of KC with ACEi use was 1.03 (95% CI 0.67–1.59). Analysis of ARB use included three studies (two case-control and one matched cohort) with 28,941 cases of skin cancer. Among ARB users, the risk of skin cancer was not significant (RR 1.41, 95% CI 0.51–3.91). Summary statistics were not reported for KC specifically.

Another meta-analysis explored the association between ACEi and ARB with type of KC [25]. For BCC, findings were imprecise for both ACEi (OR 1.50, 95% CI 0.70–3.22) and ARB (OR 1.75, 95% CI 0.68–4.49), based on two studies (one case-control and one cohort). For SCC, the findings were similar for both ACEi (OR 1.42, 95% CI 0.81–2.50) and ARB users (OR 1.54, 95% CI 0.82–2.90) based on the two studies. However, use of ACEi or ARB was associated with a significantly decreased risk of BCC (OR 0.53, 95% CI 0.39–0.71) and SCC (OR 0.58, 95% CI 0.42–0.80) based on two cohort studies among 1616 kidney transplant recipients.

##### Melanoma

In a meta-analysis, the OR for risk of melanoma was 1.08 (95% CI 0.95–1.23) for the use of ACEi or ARB including three case-control and one matched cohort study. For ARB, the OR was 1.12 (95% CI 0.95–1.31) including two case-control and one matched cohort study [24]. In another meta-analysis, the RR for ACEi was 1.23 (95% CI 0.90–1.70) based on five studies (two cohort, two case-control and one RCT) [23]. A meta-analysis evaluating the use of ACEi or ARB and the risk of ten cancer sites reported a small elevated risk of melanoma (RR 1.09, 95% CI 1.00–1.19) [50].

### 3.2. Antidiabetics

Phototoxic eruptions have been noted in patients taking certain antidiabetic medications, such as metformin, glibenclamide (also known as glyburide) and sitagliptin [17]. Metformin is a first line medication for type 2 diabetes mellitus. In addition to its glucose lowering effects, metformin preferentially inhibits cell proliferation and induces cell death in human melanoma cells, without affecting normal melanocytes [51,52,53]. Sitagliptin is the only dipeptidyl peptidase-4 inhibitor (DPP4i) with known phototoxic properties. It contains a phenyl ring, carbonyl group and three absorption peaks, two of which are within the UVC spectrum and one within the UVA spectrum [54].

#### 3.2.1. Keratinocyte Carcinoma 

Even though metformin has been studied as skin cancer treatment in clinical trials, epidemiological studies have not demonstrated decreased risk of skin cancer among metformin users [55,56]. A recent meta-analysis found that metformin use was not significantly associated with decreased risk of BCC (OR 0.75, 95% CI 0.36–1.57) or SCC (OR 0.98, 95% CI 0.06–15.60), based on three RCTs and two RCTs, respectively [57]. These authors also included a separate meta-analysis on the risk of KC based on two cohort studies with similar null findings (RR 0.65, 95% CI 0.35–1.18) [57].

There were no reported results on skin cancer risk for sitagliptin, which is the only known DDP4i with phototoxic potential. A large meta-analysis exploring the cancer risk for multiple DPP4i found no significant risk of BCC (RR 0.95, 95% CI 0.42–2.12) based on 11 RCTs [58]. Null findings were also reported for lip carcinoma based on three RCTs, while the risk of SCC was not explored. No relevant epidemiological studies have explored the risk of skin cancer from glyburide therapy.

#### 3.2.2. Melanoma

In a meta-analysis based on six RCTs, metformin use was not significantly associated with decreased risk of melanoma (OR 0.82, 95% CI 0.27–2.43) [57]. Similarly null findings on the risk of melanoma were found in meta-analysis of three cohort studies (RR 0.91, 95% CI 0.62–1.33) [57].

The meta-analysis of multiple DPP4i found no significant risk of melanoma based on 12 RCTs [58]. Again, the risk of melanoma among sitagliptin users was not reported.

### 3.3. Amiodarone

Amiodarone has been used for decades in management and prevention of severe cardiac arrhythmias, including ventricular tachycardia and atrial fibrillation. Multiple case reports have documented phototoxicity and skin cancer development in association with amiodarone use [59,60,61]. In fact, one estimate cites that 75% of patients treated with amiodarone experience photosensitivity [62]. Photosensitivity is related to cumulative dose, often occurring after continuous use of amiodarone for four months or more [62]. For patients undergoing external beam radiation therapy, concurrent amiodarone use may enhance the radiation effects on the skin and mucosa, thereby increasing the risk of secondary carcinogenesis [63,64].

#### Skin Cancer

Amiodarone use has been linked to multiple cancers, including skin cancer [65,66]. Two epidemiological studies have explored the association between amiodarone use and skin cancer overall, with no separate evaluation of KC and melanoma. In a cohort study including 6418 participants treated with amiodarone in Taiwan, only 6 participants developed skin cancer. There was no significant difference in the incidence of skin cancer among amiodarone users and among the general population [65]. Similar null results with no dose-response relationship were found in a cohort of 18,503 Danish patients with atrial fibrillation who were followed for a median of 8 years [67].

### 3.4. NSAIDs

NSAIDs are extremely common medications used for their analgesic properties. Within this class of medications, naproxen and piroxicam are non-selective cyclooxygenase (COX) inhibitors with photosensitizing potential, in addition to anti-inflammatory, anti-proliferative and pro-apoptotic effects. Naproxen has a propionic aromatic acid structure and can induce skin photosensitivity, DNA photocleavage and photo-hemolytic activity in red blood cells [68,69,70]. In a study of human fibroblasts incubated with naproxen, exposure to UVA led to a decrease in cell viability [71]. Incubating cells with naproxen also led to modification of proteins, production of ROS, phosphorylation of p38 and slowed replication of DNA [72]. On the other hand, naproxen has been shown to inhibit BCC and SCC development, reduce tumor multiplicity and decrease the number of cutaneous tumor lesions in animal studies [73,74,75,76].

In a study of five anti-inflammatory medications, incubation of piroxicam under UVA induced the formation of damaged single-stranded DNA [77]. On the other hand, piroxicam prevents tumor invasion by inhibiting angiogenesis, immunosuppression, growth factor recruitment and production of carcinogenic mediators [78,79]. Topical piroxicam has been extensively studied as a therapeutic agent for pre-malignant actinic keratoses (AK) [78,80,81,82,83]. Among hypertensive patients with multiple AK, treatment with topical piroxicam and sunscreen for six months led to significant reduction in AK count [84]. This decrease was significantly greater among hypertensive patients taking photosensitizing thiazide diuretics versus those on non-photosensitizing antihypertensive therapies.

#### 3.4.1. Keratinocyte Carcinoma

A meta-analysis including 17 studies found a RR of 0.93 (95% CI 0.87–0.99) for non-selective COX inhibitors and skin cancer overall [85]. Results were similar for BCC (RR 0.94, 95% CI 0.89–1.00) and SCC (RR 0.90, 95% CI 0.83–0.98). Selective COX-2 inhibitors were not associated with the risk of BCC or SCC.

#### 3.4.2. Melanoma

Neither non-selective COX inhibitors nor selective COX-2 inhibitors was significantly associated with melanoma [85]. The RR was 0.96 (95% CI 0.84–1.09) for non-selective COX inhibitors based on 11 studies, and was 1.04 (95% CI 0.99–1.19) for selective COX-2 inhibitors based on four studies. The included epidemiological studies did not report on individual NSAIDs, so specific results pertaining to naproxen and piroxicam could not be determined.

A case-control study in the Netherlands evaluated multiple commonly used PSMs and found that use of propionic acid derivative NSAIDs was associated with an increased risk of melanoma (OR 1.33, 95% CI 1.14–1.54) [16]. The most commonly prescribed propionic acid derivative NSAIDs include naproxen, ibuprofen and ketoprofen.

### 3.5. Statins

Statins, also known as HMG-CoA reductase inhibitors, have been instrumental in reducing morbidity and mortality for patients at high risk of cardiovascular disease [86]. Several statins are used in the US, including atorvastatin, lovastatin, pitavastatin, pravastatin, rosuvastatin and simvastatin [86]. While photosensitivity is not a common adverse effect of statins, there have been some case studies of patients who have experienced phototoxic reactions associated with statin use [87,88]. Phototoxicity of statins may be mediated by the formation of photoproducts that serve as strong photosensitizers [89,90]. In general, studies exploring the effects of statins on the risk of skin cancer have demonstrated mixed results. Some studies suggest that statins may suppress T-cell responses to malignant cells, impairing the anti-tumor immune response [91]. On the other hand, in vitro and in vivo studies have shown that certain statins may exert anti-cancer effects on melanoma cells [92,93,94].

#### 3.5.1. Keratinocyte Carcinoma

Multiple meta-analyses have investigated the relationship between statins and KC [95,96,97,98]. The most recent analysis by Yang et al. included a total of 14 RCTs with 1211 KC events [98]. No association between statin use and KC was found (RR 1.09, 95% CI 0.85–1.39). However, analysis of four observational studies (three cohort and one case-control study) with 55,793 KC cases demonstrated a small increase in risk of KC among statin users versus non-users (RR 1.11, 95% CI 1.02–1.22) [98]. Subgroup analysis showed a modest increase in risk of KC for lipophilic-statins, including atorvastatin, lovastatin and simvastatin (RR 1.14, 95% CI 1.04–1.24), and for low-potency statins, including fluvastatin, lovastatin, pravastatin and simvastatin (RR 1.14, 95% CI 1.03–1.26). Additionally, those who used statins for one year or more were at modestly increased risk of KC development compared to non-users (RR 1.14, 95% CI 1.09–1.18). A prospective study, published after the meta-analysis, analyzed two pooled cohorts and found no association between statin use and the risk of BCC or SCC [99]. However, among men, there was a significant trend towards higher BCC risk with longer duration of statin use (P-trend = 0.003).

#### 3.5.2. Melanoma

A large meta-analysis by Li et al. explored the risk of melanoma among statin users and non-users, based on 24 studies (seventeen RCTs, five case-control and two cohort) with 8433 melanoma cases [96]. There was no association between statin use and melanoma risk (RR 0.94, 95% CI 0.85–1.04). Similarly, subgroup analyses by study design, geographic location, study duration and duration of statin use did not demonstrate any significant associations. These findings are in line with prior meta-analyses [100,101,102]. Moreover, a prospective study, published after the meta-analysis by Li et al., found no association between statin use and melanoma risk based on two prospective studies [99].

### 3.6. Antibacterial Agents

Tetracyclines are a class of broad-spectrum antibiotics used to treat various infectious diseases [103]. Certain types of tetracyclines, such as doxycycline, tetracycline and chlortetracycline, are photosensitizing, leading to phototoxic dermatoses after UVA or UVB exposure [17]. The phototoxic property of these tetracyclines is due to their induction of oxidative stress in melanocytes [104,105].

Nalidixic acid and the derivative fluoroquinolone antibiotics have also been implicated in photosensitive and phototoxic reactions [17,106]. Biochemical studies have delineated the relationship between the atomic drug structure and the magnitude of potential phototoxicity. For example, certain fluoroquinolones, such as sparfloxacin and lomefloxacin, contain a halogen group in their position 8, which affords the greatest phototoxic potential [107,108,109]. Alternatively, fluroquinolones that contain a hydrogen group in position 8, such as ciprofloxacin and levofloxacin, have milder phototoxic potential [17,108]. The least phototoxic potential has been demonstrated in fluoroquinolones with a methoxy group in position 8, such as moxifloxacin [108,110]. There are a few epidemiological studies that examine associations between photosensitizing antimicrobial exposure and risk of skin cancer.

#### 3.6.1. Keratinocyte Carcinoma

A recent US-based prospective cohort study, including 36,377 cases of BCC, found a small increased risk of BCC among tetracycline users (Hazard Ratio [HR] 1.11, 95% CI 1.02–1.21), with higher BCC risk with increased duration of use (P-trend = 0.05) [111]. While the study, found no significant association with the risk of SCC (HR 1.04, 95% CI 0.91–1.18, SCC cases = 3332), positive interactions between tetracycline use and UV exposure during adulthood on SCC risk were observed (P-interaction = 0.05). The HR for SCC associated with tetracycline use was 0.89 (95% CI 0.73–1.10) for participants with lower UV exposure and 1.17 (95% CI 0.99–1.40) for those with higher UV exposure.

A Danish national register-based cohort study with 35,328 BCC cases and 6550 SCC cases found significant associations between the risks of BCC and SCC and short-term use of any photosensitizing antimicrobial (including tetracycline, doxycycline, ciprofloxacin, levofloxacin and others) [11]. Some of the medications suggested a dose-response relationship. A US-based case-control study found a significant association between use of photosensitizing antimicrobials (including tetracycline, doxycycline, trimethoprim/sulfamethoxazole and ciprofloxacin) and BCC risk (OR 1.9, 95% CI 1.3–2.8), with higher risk associated with long-term use [112]. Tetracycline use was significantly associated with BCC (OR 1.8, 95% CI 1.2–2.8), but not with SCC (OR 1.0, 95% CI 0.6–1.7).

#### 3.6.2. Melanoma

The US cohort study by Li et al. found no association between tetracycline use and melanoma (HR 1.09, 95% CI 0.94–1.27, melanoma cases = 1831) [111]. However, the Danish cohort study found a modest significant association between short-term tetracycline use and melanoma (Incidence Rate Ratio [IRR] 1.1, 95% CI 1.1–1.3) [11]. A case-control study in the Netherlands evaluated multiple commonly used PSMs and found that use of quinolones was associated with an increased risk of melanoma (OR 1.33, 95% CI 1.01–1.76) [16].

### 3.7. Voriconazole

Voriconazole is a broad-spectrum triazole antifungal medication commonly used to treat candidiasis and invasive aspergillosis. It is also prophylactically administered to immunocompromised patients to prevent invasive fungal infections [113]. Voriconazole’s primary drug metabolite, voriconazole n-oxide (VNO), may facilitate UV-induced DNA damage and inhibit DNA repair [114]. Accumulation of VNO and its UVB photoproduct within cells increases UVA sensitization and generates ROS that induce oxidative DNA damage [114,115]. Furthermore, in vitro studies on keratinocytes exposed to UV radiation demonstrate that voriconazole may have direct pro-carcinogenic effects by inhibiting catalase, which is the scavenging enzyme that eliminates free radicals [116]. Additionally, voriconazole may promote tumor development by upregulating the aryl hydrocarbon receptor-dependent COX pathway [21]. Finally, cell studies on human keratinocytes have shown that voriconazole may induce SCC by regulating specific cell cycle and terminal differentiation pathways [117,118].

#### 3.7.1. Keratinocyte Carcinoma

A meta-analysis by Tang et al. explored voriconazole use and the risk of SCC and BCC among lung transplant (LTx) and hematopoietic cell transplant (HCTx) patients [119]. Two cohort studies with only 41 BCC cases and five cohort studies with 272 SCC cases were included. There was no association between voriconazole use and risk of BCC with an RR of 0.84 (95% CI 0.41–1.71), potentially due to limited statistical power. However, there was a significant association with the risk of SCC (RR 1.86, 95% CI 1.36–2.55).

Subgroup analyses by transplant type demonstrated increased risk of SCC associated with voriconazole use in both LTx (RR 1.65, 95% CI 1.02–2.68) and HCTx patients (RR 2.29, 95% CI 1.37–3.82). Even after adjusting for sunlight and UV exposure, there was significantly increased SCC risk with voriconazole exposure. Two included studies showed a dose-response for voriconazole and SCC risk. Additionally, longer duration of voriconazole use was significantly associated with increased risk of SCC (RR 1.72, 95% CI 1.09–2.72).

Few epidemiological studies examining the association between voriconazole and KC risk have been published since the meta-analysis. A multi-center case-control study including 40 cases of BCC and 90 cases of SCC found that voriconazole exposure was associated with worse 3-year survival for KC patients and with SCC occurrence (cases: 15% versus controls: 0%) [120]. A recent retrospective cohort study of 287 LTx patients found that use of voriconazole for 100 days or more was associated with a two-fold increase in risk of SCC [121]. Another retrospective cohort study of 90 LTx patients also found that longer duration of voriconazole use served as an independent risk factor for KC (HR 1.03, 95% CI 1.00–1.06) [122]. 

#### 3.7.2. Melanoma

Some case reports have documented melanoma development in patients with chronic photosensitivity associated with long-term voriconazole therapy [123]. However, there are no epidemiological studies exploring this association.

### 3.8. Vemurafenib

Vemurafenib is a BRAF kinase inhibitor often used as a targeted therapy for late-stage and metastatic melanoma [124]. This drug is specifically used in patients with the BRAF V600E mutation, which is found in approximately 60% of melanomas [124]. A phase III clinical trial demonstrated an increased survival rate among melanoma patients treated with vemurafenib compared to those treated with dacarbazine [125]. While vemurafenib has shown great efficacy, the phototoxic effects of this medication may lead to the development of localized SCC [126]. SCC development in patients taking vemurafenib may be mediated by activation of the MAPK pathway [126]. A multi-center, international, open-label safety study enrolled 3226 patients with advanced metastatic BRAF V600 melanoma and investigated vemurafenib’s effectiveness [127]. Adverse effects were documented, such as the development of SCC (12%), rashes (5%) and photosensitivity (2%) [127].

#### Keratinocyte Carcinoma

A recent meta-analysis of 11 clinical trials (including open phase, randomized and general clinical trials) evaluated dermatological toxicities, such as rashes, photosensitivity reactions, SCC and keratoacanthoma, associated with vemurafenib use among melanoma patients [128]. Vemurafenib use was associated with increased SCC prevalence of 18% (95% CI 12–26%). In three included trials, vemurafenib use was associated with high-grade SCC, with an overall prevalence of high-grade SCC of 16% (95% CI 11–23%). The overall prevalence for photosensitivity reactions among melanoma patients using vemurafenib was 30% (95% CI: 23–38%).

### 3.9. PSMs

Only a few studies evaluated the use of any PSM in relation to skin cancer risk. A population-based case-control study in New Hampshire with 1567 BCC and 1,599 SCC cases revealed a risk of SCC (OR 1.2, 95% CI 1.0–1.4) and BCC (OR 1.2, 95% CI 0.9–1.5) associated with use of any PSM, although not statistically significant [112]. However, there was an increased risk of BCC among PSM users who were diagnosed with BCC before they were 50 years old (OR 1.5, 95% CI 1.1–2.1). Among PSM users with a fair skin phenotype, the risk of SCC was increased (tendency to sunburn: OR 1.5, 95% CI 1.1–2.0; tendency to tan: OR 1.0, 95% CI 0.8–1.3). Another study based in Denmark found that both short term and long-term use of 19 PSMs were associated with increased risks of BCC, SCC and melanoma with RRs ranging from 1.11–1.47 [11]. Only two PSMs were associated with a ≥20% increased risk of skin cancer, with elevated risk related to longer duration of use.

In the previously mentioned Netherlands case-control study that demonstrated over 40% of the population used PSMs, Siiskonen et al. also found that propionic acid derivative NSAIDs and quinolones were associated with increased risk of melanoma [16]. In a prospective cohort study of hypertensive non-Hispanic white patients in California, antihypertensive drugs were categorized as photosensitizing (alpha-2 receptor agonists and diuretics), non-photosensitizing (alpha-blockers, beta-blockers, central agonists and ARB) or unknown (ACEi, CCBs and vasodilators) [129]. The authors found that users of photosensitizing and unknown antihypertensives were at increased risk of SCC, compared with non-users, who did not use antihypertensive drugs after cohort entry [129]. However, there was no association with use of non-photosensitizing antihypertensives, compared with non-users [129].

## 4. Discussion

This review describes findings from epidemiological studies that explore use of PSMs and the risk of skin cancer. Evidence is summarized for common PSMs, including antihypertensives, antidiabetics, amiodarone, NSAIDs, statins, antibacterial agents, voriconazole and vemurafenib, that have at least one epidemiological study investigating their association with skin cancer.

Of the common PSMs reviewed, the antihypertensive medications were most extensively investigated. Each antihypertensive medication class was explored in at least one meta-analysis. These meta-analyses revealed that diuretics were associated with increased risks of all skin cancer types, while CCBs and beta-blockers were only associated with certain types of skin cancer (Table 2). For ACEi and ARB, there were suggestions of a non-significant positive direction of association with both KC and melanoma. On the other hand, certain beta-blockers may have beneficial therapeutic anti-cancer properties [37,38,43]. One study of beta-blockers and melanoma survival provided evidence in support of the beneficial role that certain beta-blockers may play in cancer care [42].

The relationship between statins and skin cancer was also extensively evaluated in multiple meta-analyses, relative to other PSMs. There was a modest positive association between statin use and KC in observational studies, but null findings in RCTs [98].

There were a few studies on metformin and amiodarone that demonstrated no association with skin cancer. Even though only a few studies are available, the literature suggests a positive association between antibacterial agents and skin cancer. In the antifungal category, voriconazole was associated with increased risk of SCC, but not BCC. There were no epidemiological studies of voriconazole use and melanoma risk. Vemurafenib, a melanoma treatment option, was associated with increased risk of SCC.

For some medications, such as naproxen, piroxicam and sitagliptin, there were no epidemiological studies that focused on skin cancer risk associated with exposure to the individual PSM. Instead, meta-analyses reported on drug classes, combining medications with and without photosensitizing properties. There were no epidemiological studies of other common PSMs, including glyburide, chlorpromazine, thioridazine and nalidixic acid.

In addition to beta-blockers, there are other PSMs with properties that may afford skin cancer protection. These include metformin, naproxen and piroxicam. In fact, metformin has been evaluated in several clinical trials as a chemopreventive agent for patients with melanoma. Piroxicam may even mitigate the phototoxic effects associated with thiazide use. The potential carcinogenic effects of these PSMs may be balanced by their anti-cancer properties, attenuating the risk of skin cancer associated with use.

We identified several research gaps in the literature. First, a limited number of studies evaluated the dose and duration of PSMs and the risk of skin cancer [26]. Unfortunately, evaluation of ever versus never use of PSMs provides limited information with no differentiation between short-term and long-term use. More studies evaluating the dose and duration of PSM use to delineate dose-response effects may further strengthen associations and suggest a causal link between PSMs and skin cancer.

Second, nearly all studies evaluated the association between use of a single PSM and skin cancer [11,112]. However, in practice, numerous PSMs from various drug classes are often taken simultaneously. Polypharmacy is particularly common in elderly patients with multiple comorbidities [130,131]. Since increasing age is also a known risk factor for skin cancer, it is essential to determine the effects of PSM polypharmacy in this susceptible patient population.

Third, for certain conditions, such as hypertension, many of the therapeutic drug options are potentially photosensitizing [23]. In this case, evaluating skin cancer risk associated with one medication may be insufficient because those who do not use the specific medication may use another antihypertensive medication that also has phototoxic properties. To our knowledge, no study has evaluated different types of antihypertensive medications (using one common reference group) in relation to skin cancer.

Fourth, many previous studies were based on cancer registries with limited demographic information and almost no data on skin cancer risk factors such as constitutional factors and sun exposure [18,23,26]. Thus, the studies were often unable to adjust for skin cancer risk factors. In addition, evaluating the interaction with skin cancer risk factors may deepen our understanding of the relationship between PSMs and skin cancer. For example, there may be increased risks of skin cancer among PSM users with sun-sensitive phenotypes [112].

Fifth, skin cancers related to PSMs could occur on sun-exposed body sites more frequently than on sun-shielded body sites. However, only a few studies on PSMs evaluated the risk of skin cancer based on tumor body site.

Sixth, studies relying on prescription databases may not know whether filled prescriptions were actually used by the patients and may lead to misclassification of some non-users as users, potentially biasing results towards the null [11,18].

Seventh, there is also an issue of confounding by indication: PSM users may be at increased risk of skin cancer, due to their underlying disease, rather than their medication use. For example, since antifungal medications are more commonly used in immunocompromised patients such as transplant recipients, it is possible that the immunosuppressed state is driving the association with skin cancer, rather than the antifungal medication. However, many of the diseases included in this review such as hypertension and cardiac arrhythmias are not known risk factors of skin cancer.

Finally, individuals taking PSMs may see doctors more frequently, resulting in a higher chance of detection and diagnosis of skin cancer during office visits [132]. Many of the previous studies lacked an active comparator medication, making detection bias and confounding by indication more potentially problematic. More studies evaluating multiple PSMs individually and together would provide a better understanding of the overall impact of PSMs on skin cancer risk.

## 5. Conclusions

In summary, there are several commonly used PSMs, which are associated with elevated risk of skin cancer based on review of epidemiological literature. Still, these studies often lacked adjustment for skin cancer risk factors and evaluation of dose-response relationships. There was limited evaluation of the interaction with skin cancer risk factors. Additionally, there is a lack of epidemiological studies on skin cancer risk associated with many PSMs. Delineating the effects of concurrent use of multiple PSMs is necessary since they are often taken in tandem with one another.

It is important to note that while the reported RR of skin cancer associated with PSMs, which represent the strength of the associations, may be moderate or small, use of PSMs is very common, especially among older populations who are more susceptible to skin cancer development [15,16]. In addition, skin cancer is the most common cancer in the US [1]. Thus, even small increases in RRs may have high absolute risks and have huge clinical and public health implications [133]. Addressing the research gaps outlined in this review should be prioritized, because as the US population ages, the number of patients prescribed PSMs will likely continue to rise.

## Figures and Tables

**Table 1:** Common photosensitizing medications.

Class	Medications
Antihypertensives	Diuretics
	CCBs
	Beta-blockers
	ACEi
	ARB
Antidiabetics	Metformin
	Sitagliptin
	Glyburide
NSAIDs	Naproxen
	Piroxicam
Antibacterial agents	Tetracycline
	Doxycycline
	Ciprofloxacin
	Levofloxacin
Others	Amiodarone
	Statins
	Vemurafenib
	Voriconazole

**Table 2:** Summary of the association between photosensitizing medications and the risk of skin cancer ^1^.

Drug Class/Medications	BCC	SCC	Melanoma
Diuretics	+ (4)	+ (7)	+ (5)
CCBs	+ (3)	Null (3)	+ (5)
Beta-Blockers	+ (3)	Null (3)	Null (3)
ACEi	Null (2)	Null (2)	Null (5)
ARB	Null (2)	Null (2)	Null (3)
Metformin	Null (3)	Null (2)	Null (9)
Amiodarone	Null (2)		
Statins	Null (20)		Null (26)
Voriconazole	Null (2)	+ (6)	0
Vemurafenib	0	+ (11)	0
Antibacterial agents	+ (3)	+ (3)	+ (3)

^1^ Included medications are those with more than two epidemiological studies. Direction of association: +,-, null (total number of studies described, composite of those included in meta-analyses and individual studies). The cell is marked 0 for medications where no relative epidemiological studies were found.

## Data Availability

Primary data cited in this review is openly available in PubMed.
